# Protein PGLYRP1/Tag7 Peptides Decrease the Proinflammatory Response in Human Blood Cells and Mouse Model of Diffuse Alveolar Damage of Lung through Blockage of the TREM-1 and TNFR1 Receptors

**DOI:** 10.3390/ijms222011213

**Published:** 2021-10-18

**Authors:** Tatiana N. Sharapova, Elena A. Romanova, Aleksandr S. Chernov, Alexey N. Minakov, Vitaly A. Kazakov, Anna A. Kudriaeva, Alexey A. Belogurov, Olga K. Ivanova, Alexander G. Gabibov, Georgii B. Telegin, Denis V. Yashin, Lidia P. Sashchenko

**Affiliations:** 1 Laboratory of Molecular Immunogenetics of Cancer, Institute of Gene Biology RAS, Vavilova 34/5, 111394 Moscow, Russia; elrom4@rambler.ru (E.A.R.); olga.k.ivanova@gmail.com (O.K.I.); yashin_co@mail.ru (D.V.Y.); sashchenko@genebiology.ru (L.P.S.); 2 Branch of Shemyakin and Ovchinnikov Institute of Bioorganic Chemistry of the Russian Academy of Sciences, 142290 Pushchino, Russia; alexandrchernov1984@gmail.com (A.S.C.); minakov@bibch.ru (A.N.M.); vitalij.tomsk@list.ru (V.A.K.); telegin@bibch.ru (G.B.T.); 3 Shemyakin and Ovchinnikov Institute of Bioorganic Chemistry, Russian Academy of Sciences, 117997 Moscow, Russia; anna.kudriaeva@ibch.ru (A.A.K.); belogurov@ibch.ru (A.A.B.J.); gabibov@ibch.ru (A.G.G.)

**Keywords:** cytokines, Tag7, acute lung injury, TREM-1, TNFR1, COVID-19

## Abstract

Infection caused by the severe acute respiratory syndrome coronavirus (SARS-CoV-2) in many cases is accompanied by the release of a large amount of proinflammatory cytokines in an event known as “cytokine storm”, which is associated with severe coronavirus disease 2019 (COVID-19) cases and high mortality. The excessive production of proinflammatory cytokines is linked, inter alia, to the enhanced activity of receptors capable of recognizing the conservative regions of pathogens and cell debris, namely TLRs, TREM-1 and TNFR1. Here we report that peptides derived from innate immunity protein Tag7 inhibit activation of TREM-1 and TNFR1 receptors during acute inflammation. Peptides from the N-terminal fragment of Tag7 bind only to TREM-1, while peptides from the C-terminal fragment interact solely with TNFR1. Selected peptides are capable of inhibiting the production of proinflammatory cytokines both in peripheral blood mononuclear cells (PBMCs) from healthy donors and in vivo in the mouse model of acute lung injury (ALI) by diffuse alveolar damage (DAD). Treatment with peptides significantly decreases the infiltration of mononuclear cells to lungs in animals with DAD. Our findings suggest that Tag7-derived peptides might be beneficial in terms of the therapy or prevention of acute lung injury, e.g., for treating COVID-19 patients with severe pulmonary lesions.

## 1. Introduction

Cytokines are multifunctional proteins playing a main role in the innate immune response. Moreover, proinflammatory cytokines are equally crucial during the activation of the immune system. However, the overproduction of proinflammatory cytokines associated with different alterations in the immune response can cause severe negative consequences such as tissue destruction in autoimmune arthritis or the development of septic shock and acute respiratory distress syndrome (ARDS) [1,2,3]. In many cases, the infection caused by SARS-CoV-2 is accompanied by the release of a large number of proinflammatory cytokines in an event known as “cytokine storm”. A too strong proinflammatory response seen in cytokine storm is similar to septic shock as regards the inducing mechanisms. It also contributes to the severe clinical course and high fatality rates in patients infected with the SARS-CoV-2 and Ebola viruses [4].

One of the causes of the excessive production of proinflammatory cytokines is linked to the enhanced activity of the pathogen recognition receptors (PRRs), capable of recognizing pathogen-associated molecular patterns (PAMPs) and damage-associated molecular patterns (DAMPs). PRRs include a set of toll-like receptors (TLRs), which play crucial roles in the innate immune system. TLRs can interact with various ligands derived from both PAMPs and DAMPs. Activation of TLRs leads to the NF-kB-induced expression of genes of proinflammatory cytokines and receptors on the cell surface, including triggering receptor expressed on myeloid cells-1 (TREM-1) [5,6].

TREM-1 is important as an amplifier of the inflammatory response. The described ligands of TREM-1 include the high mobility group box 1 protein (HMGBI) nuclear protein and the innate immune protein PGLYRP1 (Tag7) [7,8,9]. Our recent study has demonstrated that Hsp70, the major heat shock protein, can be also considered as another ligand for TREM-1 [10]. In turn, TREM-1 is expressed on monocytes, neutrophils, antigen-presenting cells and NK cells, as well as selective T- and B-lymphocyte populations, epithelial cells and fibroblasts. TREM-1 activation is known to induce the release of proinflammatory cytokines and contribute to proinflammatory effects linked to the activation of other innate immunity receptors, e.g., TLR4 [11].

The inflammation might also be induced by tumor necrosis factor receptor 1 (TNFR1), which does not belong to the family of innate immunity receptors. The receptor TNFR1 is expressed universally in multiple tissues, including immune cells. This receptor is capable of inducing cell death via apoptosis or necroptosis and triggering the NF-kB-mediated activation of proinflammatory cytokine genes [12].

Thus, the above-mentioned receptors are responsible for the expression of proinflammatory cytokine genes. A proposed option for decreasing receptor sensitivity is to identify biologically active fragments of the regulatory proteins that can prevent the binding of these receptors to ligands. Though TLR4 and TREM-1 peptide inhibitors have been described, the expansion of a spectrum of these protein fragments may help to determine the optimal conditions for reducing inflammation.

The innate immune protein PGLYRP1 (Tag7) can bind to the receptors TREM-1 and TNFR1. Our study has demonstrated that Tag7 binding to TREM-1 leads to the secretion of proinflammatory cytokines which in turn triggers the induction of antitumor activity against HLA-negative cancer cells in human peripheral blood cells [13]. Tag7 binding to TNFR1 inhibits TNFα-induced death of tumor cells [14,15]. We investigated Tag7 primary sequence fragments responsible for binding to these receptors. A 12-mer peptide 17.1 and its shortened fragment 17.1a, located in the C-terminal of the Tag7 molecule, interact with TNFR1. Both peptides inhibited TNFα-mediated cell death and slowed the progression of CFA-induced autoimmune arthritis. The preliminary results suggest that the TREM-1 binding region is located in the N-terminal of the Tag7 molecule [16,17]. An assumption can be made that these Tag7 fragments might be involved in the inflammatory response regulation. After binding to TREM-1 or TNFR1, they prevent the interaction between these receptors and their ligands and reduce the levels of proinflammatory cytokines. As a result, they can mitigate such an excessive immune response as cytokine storm syndrome in patients with SARS-CoV-2.

The objective of this study was to identify a new Tag7 peptide that interacts with TREM-1 and to characterize the involvement of the protein fragments located on different polypeptide chain terminals in the regulation of gene expression, the production of proinflammatory cytokines and the protective action against inflammation progression using human blood cells (PBMCs) and a mouse model of acute lung injury by DAD.

## 2. Results

### 2.1. Tag7 Protein N-Terminal Peptides Interact with the Receptor TREM-1 and Inhibit the Cytotoxic Activity of PBMCs

The preliminary results of our previous studies demonstrated that the Tag7 molecule N-terminal fragment interacted with TREM-1. In the present study, we have identified a peptide that inhibits the interaction between Tag7 and this receptor. To achieve this goal, we used a limited proteolysis by trypsin and selected experimental conditions that allow obtaining the optimal low-molecular-weight peptides for analysis and further synthesis.

**Figure 1:**
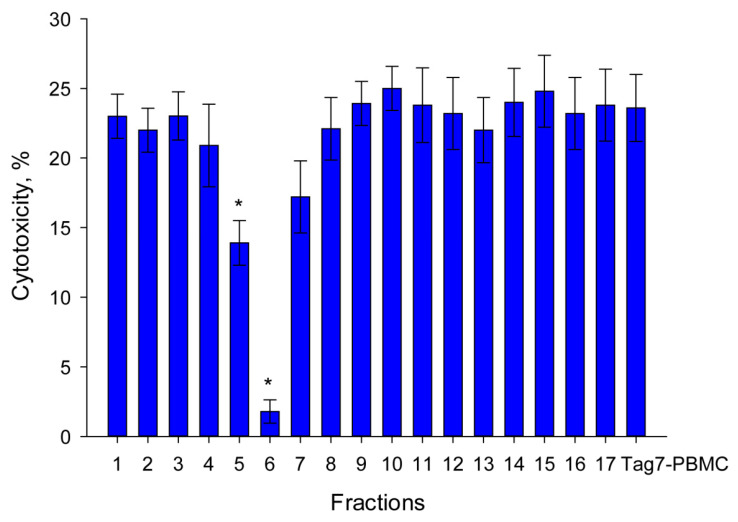
Inhibition by peptide fractions after hydrolysis and separation by chromatography using a Superdex Peptide column of lymphocyte activation for 6 days by Tag7 protein. An aliquot of each fraction was added to PBMC 30 min prior to the addition of Tag7 protein. The cytotoxic activity of lymphocytes was measured against K562 cells (one-way ANOVA, *p*-value: * < 0.05 vs. Tag7-PBMC).

After a five-hour hydrolysis, the mixture of peptides was separated by gel filtration on a Superdex Peptides column, and the inhibiting activity of each fraction was determined. Aliquots of each fraction were applied to the PBMCs before treatment with Tag7. Following six-day incubation, PBMC cytotoxic activity against HLA-negative tumor cells was detected (Figure 1).

Only 1 (No. 6) of 17 analyzed fractions contained a peptide that blocked the induction of PBMC cytotoxic activity (Figure 1). Using MALDI analysis, an 8-mer peptide of this fraction that corresponded to the N-terminal amino acid sequence (amino acids 77–86) was identified.

**Figure 2:**
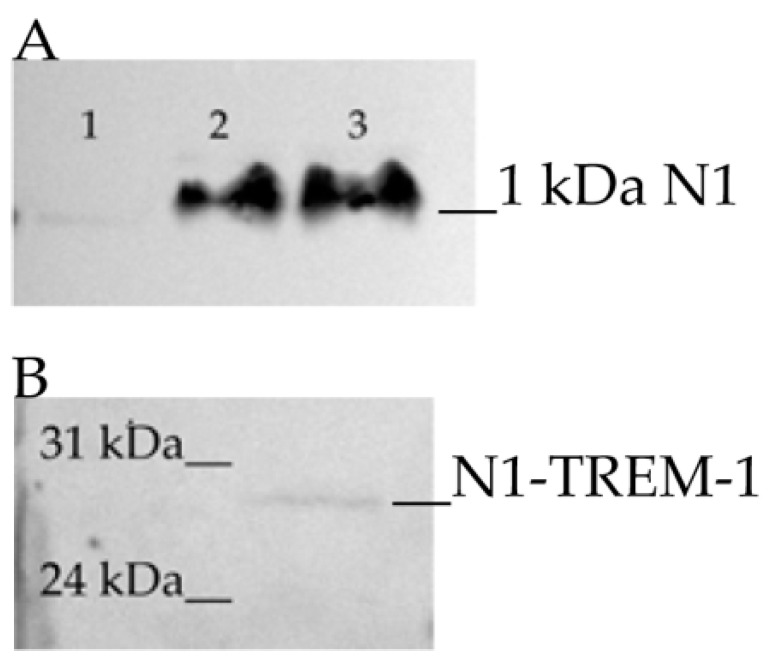
Binding of N1 with sTREM-1. (**A**) Binding of biotin-N1 with sTREM-1, immobilized on CNBr-activated Sepharose; 1—washing before elution, 2—elution of N1 peptide, 3—control N1 peptide. The resulting material was analyzed with tricine–SDS-PAGE followed by streptavidin–HRP Western blotting. (**B**) Binding of biotin-N1 with sTREM-1 on monocytes. Monocytes incubated with biotin-N1 and treated with crosslinker BS^3^. Cells lysed and purified with Dynabeads, conjugated with anti-TREM-1 antibodies. The resulting material was analyzed by SDS electrophoresis followed by streptavidin–HRP Western blotting.

The peptide was synthesized and biotinylated. At the synthesis stage, it was named N1. Then, its capability to interact with soluble sTREM-1 exodomain of the receptor TREM-1 was assessed using affinity chromatography. The material was resolved using tricine–SDS-PAGE followed by streptavidin–HRP Western blotting. It is possible to observe that the biotinylated peptide N1 binds to sTREM-I immobilized on CNBr-activated Sepharose (Figure 2A, Supplementary Materials Figure S1).

In addition, the peptide binding with sTREM-1 expressed on the cell membrane was investigated. Isolated monocytes were incubated with the biotinylated peptide N1 in the presence of a crosslinker BS^3^. Cell lysate was purified using specific anti-TREM-1 antibodies conjugated with magnetic beads (Dynabeads). The bound material was analyzed by SDS electrophoresis followed by streptavidin–HRP Western blotting. The antibody-bound fraction contained biotinylated material, and the corresponding molecular weight of TREM-1 with peptide was 27 kDa (Figure 2B, Supplementary Materials Figure S2). These findings suggest that the antibodies were bound by the TREM-1–N1 complex stabilized with BS^3^ crosslinker reagent.

**Figure 3:**
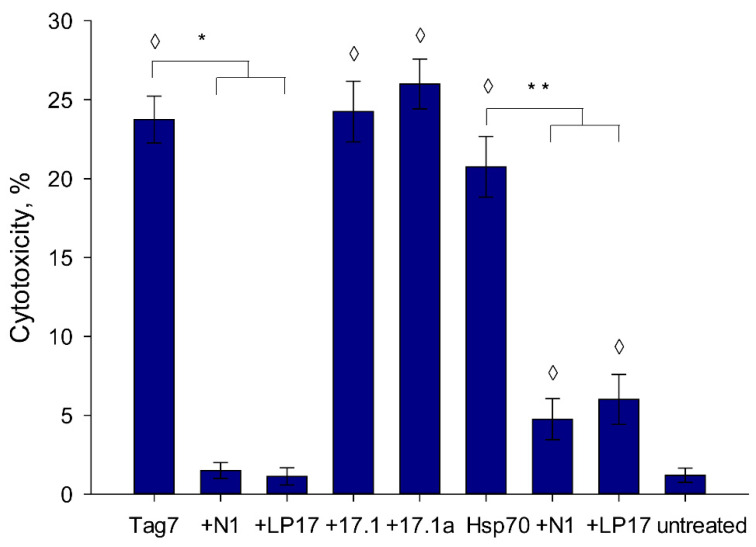
The cytotoxic activity of PBMC incubated with Tag7 or Hsp70 for six days in presence of N1, 17.1, 17.1A and LP17 peptides. Peptides were added to PBMC 30 min prior to activation. The cytotoxic activity of lymphocytes was measured against K562 cells (one-way ANOVA, *p*-value ◊ < 0.05 vs. untreated; one-way ANOVA, *p*-value: ** < 0.05, * < 0.01).

We evaluated the inhibiting activity of the synthesized peptide and came to the conclusion that N1 inhibits the capability of TREM-1 to induce the activation of cytotoxic lymphocytes similarly to the previously described LP17 inhibitor. Earlier investigated peptides 17.1 and 17.1.A capable of binding specifically to the TNFR1 receptor did not exhibit inhibiting effect (Figure 3). It should be noted that N1 reduced the cytotoxic activity initiated both by the full-length Tag7 and the major heat shock protein Hsp70 which can be also considered as a ligand for TREM-1 [10].

Thus, the peptide may inhibit the activation of cytotoxic lymphocytes which kill the HLA-negative tumor cells.

### 2.2. Protein Tag7 Peptide Fragments Binding Specifically to TREM-1 or TNFR1 Inhibit the mRNA Expression of Proinflammatory Cytokines

Once it was shown that Tag7 peptide fragment is involved in the regulation of cytotoxicity of noncanonical effector lymphocyte subpopulations in antitumor immunity, it appeared feasible to investigate its impact on the production of proinflammatory cytokines regulating the development of adaptive immunity.

To induce the gene expression of proinflammatory cytokines, we used the lipopolysaccharide (LPS) belonging to PAMPs and serving as a ligand for the receptor TLR4. Human PBMCs were used as a source of cytokines.

As mentioned above, TLR activation in the course of the persistent systemic inflammation process is frequently accompanied by activation of the receptors TREM-1 and TNFR1. Therefore, it was important to figure out which receptors were activated by PBMC treatment with LPS under the experimental conditions. For this purpose, PBMCs were preincubated with the receptor inhibitors and then treated with LPS for 12 h. We subsequently used RT-PCR to measure the changes in the mRNA level of proinflammatory cytokine IFNγ.

**Figure 4:**
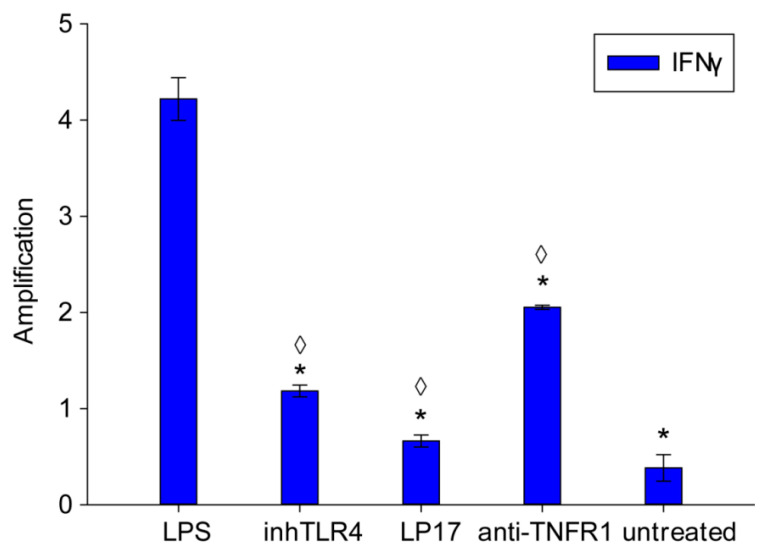
Changes in mRNA expression of IFNγ in PBMCs treated with LPS blocking of TLR4, TREM-1 and TNFR1 receptors. PBMCs incubated with LPS (100 ng/mL) only or in presence of inhTLR4, LP17 and anti-TNFR1 antibodies for 12 h. All values were normalized to control (before any treatment). Level of mRNA was measured by RT-PCR with RPLP0 and GADPH as references (one-way ANOVA, *p*-value ◊ < 0.05 vs. untreated; one-way ANOVA, *p*-value * < 0.01 vs. LPS).

As one can see, LP17, a specific TLR4 inhibitor, and anti-TNFR1 antibody markedly decrease the mRNA expression of this cytokine (Figure 4). Thus, the receptors TREM-1 and TNFR1, in addition to TLR4, contribute to the induction of the gene expression of proinflammatory cytokines.

The next step was to assess the inhibiting effect of Tag7 N- and C-terminal peptides binding specifically to TREM-1 and TNFRI. Please refer to Supplementary Materials Figure S3 to see the 3D picture of Tag7 and fragments used in this study. It shows that peptide N1 is part of the α-helix of the polypeptide chain N-terminus, not overlapping it. Therefore, an adjacent N2 peptide was synthesized, which is also part of the same α-helix. Peptide 17.1 and its shortened fragment (17.1a) were located on the C-terminus.

**Figure 5:**
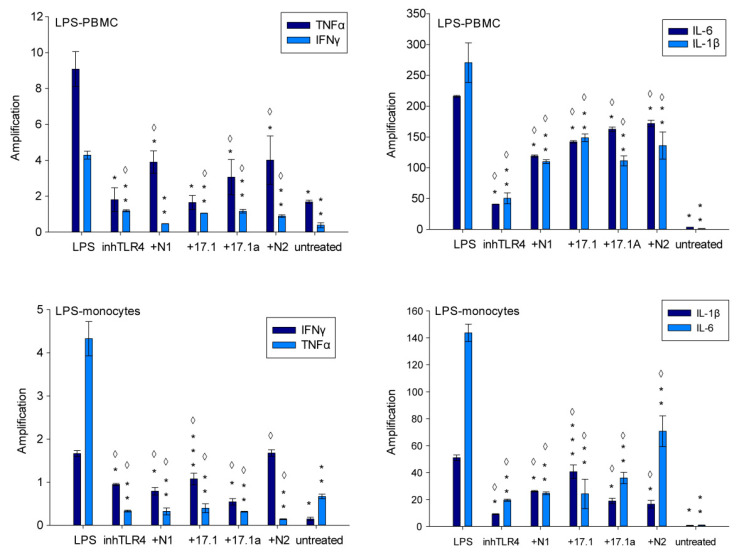
mRNA expression of IFNγ, TNFα, IL-1β and IL-6 in PBMCs or monocytes treated with LPS. Cells were incubated with LPS (100 ng/mL) only or in presence of TLR4 inhibitor (inhTLR4); peptides: N1, N2, 17.1, 17.1A for 12 h. All values were normalized to control (before any treatment). Level of mRNA measured by RT-PCR, using RPLP0 and GADPH as references. (one-way ANOVA, *p*-value ◊ < 0.05 vs. untreated; *t*-test, *p*-value *** < 0.005; one-way ANOVA, *p*-value: ** < 0.05, * < 0.01 vs. LPS).

PBMCs were preincubated with peptides N1, N2, 17.1 and 17.1a and TLR4 inhibitor (control) and then treated with LPS for 12 h. Next, changes in the mRNA levels of proinflammatory cytokines IL-6, IL-1β, TNFα and IFNγ were measured using RT-PCR. As shown, after LPS treatment, the mRNA expression of cytokines IL-6 and IL-1β was higher than the mRNA expression of cytokines TNFα and IFNγ, while preincubation with Tag7 peptide fragments resulted in a substantial decrease in the mRNA levels of studied cytokines (Figure 5).

In view of the fact that the receptors TLR4, TREM-1 and TNFR1 might be expressed on monocytes initiating the onset of immune response, these findings were confirmed by the results of LPS incubation with isolated monocytes. Furthermore, we observed a higher mRNA expression of cytokines IL-6 and IL-1β, and on top of that, the investigated peptides inhibited the gene expression of proinflammatory cytokines (Figure 5).

To summarize, peptides N1 and N2 interacting with TREM-1, as well as peptides 17.1 and 17.1A capable of recognizing the receptor TNFR1, inhibit the gene expression of proinflammatory cytokines in innate immune cells controlling inflammatory response, which may reduce inflammation.

### 2.3. Tag7 Peptides Change the Cytokine Levels in the Blood Plasma of Mice with Induced DAD

**Figure 6:**
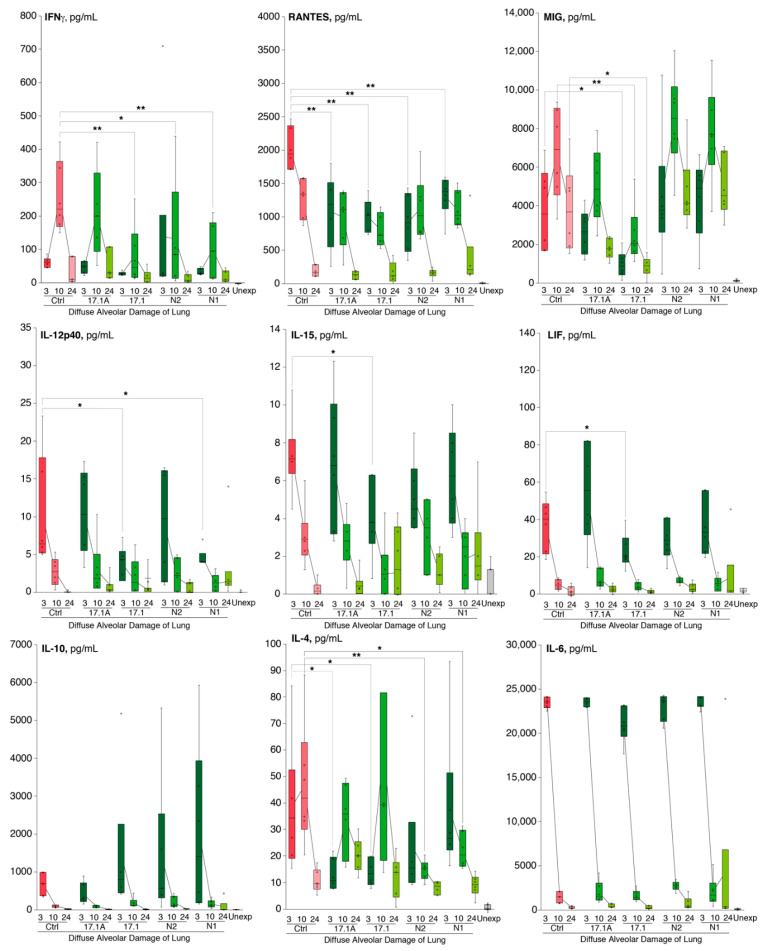
Administration of Tag7-derived peptides counteracts cytokine storm during diffuse alveolar damage of lung induction. Level of plasma cytokines in mice with DAD (red bars) after 3, 10 and 24 h after syndrome induction in comparison with unexposed animals (grey bars) and animals treated with Tag7 peptides (green bars). Bars represent median with interquartile range; 95% confidence interval is shown. Leukemia inhibitory factor (LIF); monokine induced by gamma (MIG/CXCL9); interferon-gamma (IFNγ); regulated upon activation, normal T cell expressed and secreted (RANTES/CCL5). Asterisks indicate statistically significant differences between treated and untreated animals with DAD at respective timepoints. All animals with DAD have dramatically increased levels of analyzed cytokines and chemokines in comparison with unexposed animals regardless of treatment (one-way ANOVA, *p*-value: ** < 0.05, * < 0.01).

We next measured cytokine profile in nontreated mice with induced DAD in comparison with animals treated with Tag7-derived peptides (Figure 6). Our data suggest that injection of peptide 17.1 decreased the level of plasma IFNγ by up to 5 times in comparison with nontreated animals. Peptides N1 and N2 also decreased the level of IFNγ, with less pronounced effectivity. All tested Tag7 peptides significantly decreased the level of plasma IL-4 after 3 or 10 h after DAD induction. Peptide 17.1 also decreased the level of plasma IL-15 and IL-12p40. Importantly, this peptide markedly decreased the level of chemokines monokine induced by gamma (MIG/CXCL9); leukemia inhibitory factor (LIF); and regulated upon activation, normal T cell expressed and secreted (RANTES). We also observed a tendency of the plasma IL-10 to increase in response to administration of peptides 17.1, N1 and N2. The level of other measured plasma cytokines, including IL-6 and TNFα, was not changed in treated mice in comparison with nontreated animals with DAD.

### 2.4. Tag7 Peptides Show Anti-Inflammatory Activity in a Mouse Model of DAD

The in vivo experiments included a comparative analysis of the efficacy of peptides (fragments of Tag7 protein) in the therapy of diffuse alveolar damage of the lung. A mouse model of DAD, at days 7, 14 and 30, gave the following findings: a gradual escalation of inflammatory response in the lungs that was visualized mostly as focal perivascular and peribronchial infiltration by the mononuclear cells, as well as the infiltration of alveolar walls and ducts by the mononuclear cells. The key predictor of unfavorable outcome in inflammatory response is active diapedesis of mononuclear cells to the surrounding tissues.

**Figure 7:**
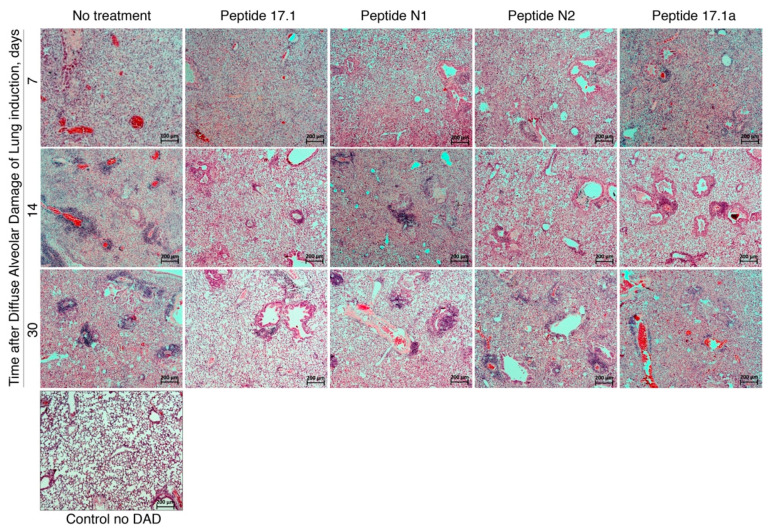
Fragments of the left lung lobe of male ICR mice, intact, on the 7th, 14th and 30th days after induction of DAD in presence of peptides 17.1, N1, N2 and 17.1A or without treatment.

All tested peptides on different days of observation demonstrated a varying anti-inflammatory effect (Figure 7). At the early stage (day 7), peptides N1 and N2 had the highest anti-inflammatory effect on the focal perivascular and peribronchial infiltration by the mononuclear cells, which was evaluated with a semiquantitative scoring system (Table 1).

In a midterm evaluation (day 14), the perivascular infiltration with the mononuclear cells was less pronounced in the group of animals treated with peptides vs. the control group of animals with saline solution. When scoring criteria were used for the evaluation of pathomorphological signs, the most beneficial results were obtained after the use of tested peptide 17.1 in the late stage of DAD (day 30), especially as regards the extent of peribronchial infiltration and a higher involvement of the left lung lobes in the systemic gas exchange (Table 1).

The findings suggest that short peptides 17.1, N1 and N2, as well as innate immunity Tag7 protein fragments, might be used in the future for effective treatment or prevention of DAD.

## 3. Discussion

In this study, the following goals were achieved: (1) A new peptide of innate immunity protein Tag7 was identified; N1 is located on the molecule N-terminus, capable of binding to the receptor TREM-1 and inhibiting its activation. (2) A new function of N-terminal peptides and the previously described C-terminal peptides of Tag7 was elucidated, i.e., their capability to inhibit the expression of proinflammatory cytokines in human immune cells ex vivo and in a mouse model of ALI in vivo. (3) It was shown that the administration of the studied peptides to mice with induced ALI alleviated the severity of pathological processes.

We have demonstrated that the Tag7 molecule has two sites that bind to its receptors. C-terminal regions of Tag7 named 17.1 and 17.1a (a shorter variant) bind to TNFR1. The Tag7 molecule N-terminal region contains a site that binds to another receptor—TREM-1. Peptides N1 and N2 derived in this study interacted with this receptor and inhibited its activation.

The study elucidated a new function of these peptides—the capability to decrease the expression of cytokines TNFα, IFNγ, IL-6 and IL-1b in PBMCs. These cytokines regulate the development of persistent inflammatory process associated with a severe clinical course. In patients with COVID-19 who have severe illness, high levels of cytokines IL-1, IL-2, IL-6, IL-7, IL-10, G-CSF, IP-10, MCP-1, MIP-1α and TNFα have been frequently reported [18]. High levels of these cytokines may lead to a “cytokine storm”, playing a key role in the pathogenesis of viral infection, the development of sepsis in response to such infection and the lung injury induced by the inflammatory process. Viral pneumonia occurring in most of these patients can be followed by acute respiratory distress syndrome or even multiple organ failure [19,20,21,22].

As mentioned above, at least three receptors, TLR4, TREM-1 and TNFR1, are responsible for the overexpression of genes of proinflammatory cytokines. Activation of each of them, as a single receptor, does not cause persistent inflammation. The receptors TREM-1 and TNFR1 are considered inflammatory signal enhancers since the simultaneous activation of these receptors leads to a dramatic increase in the production of proinflammatory cytokines. A connection between the activation of the two receptors TLR4 and TREM-1 was demonstrated [23]. They have common ligands, in particular protein HMGB1. Furthermore, the intracellular pathways of activation of the transcription factors regulating gene expression might overlap [24]. A TREM-1 gene knockdown initiates the downregulation of multiple genes involved in TLR4 signaling [25]. Viral infection can also be accompanied by the development of a systemic inflammation resulting from the overexpression of proinflammatory cytokines. The Ebola virus envelope glycoprotein (GP) is considered a ligand for TREM-1, and the interaction of this protein with TREM-1 on the neutrophils is considered a cause of cytokine storm and high mortality rates among infected individuals [26]. Recently, it has been demonstrated that a high concentration of the soluble receptor sTREM-1 in the blood correlates with severe clinical course in patients with SARS-CoV-2 [27]. TLR4 activation by its ligand triggers the activation of TNFR1 and is followed by the expression of proinflammatory cytokines [28].

Our study has demonstrated that three receptors were activated by LPS. It appears that the activation is a two-stage process. First, the activation of TLR4 and the related receptor TREM-1 stimulates the production of proinflammatory cytokines. Then, the secreted TNFα activates TNFR1, and this process is followed by the induction of gene expression of all proinflammatory cytokines. We also found that Tag7 peptide fragments bind specifically to TREM-1 and TNFR1, thus causing a dramatic drop in the mRNA levels of TNFα, IFNγ, IL-6, and IL-1b in human immune cells regulating inflammatory signal.

Originally, the terms acute lung injury (ALI) and ARDS were used interchangeably; however, considering the broad interpretation of ALI in practical medicine, it was recommended to abandon this term in clinical practice [29]. Our model permits the formation of unilateral severe diffuse alveolar damage (DAD) of the lung that serves as a morphological foundation of ARDS. ARDS occurs as a final stage of the fatal lung injury induced by viral or bacterial pneumonia, sepsis, life-threatening injury and coronaviral infection [30,31]. DAD is characterized by diffuse alveolar damage and massive infiltration with neutrophils, endothelial function disruption, microthrombosis, interstitial and alveolar edema and the overproduction of proinflammatory cytokines [32]. So far, no medications are available for the efficacious treatment or prevention of DAD or ARDS, and therefore the research in this area should be continued. Our study included a comparative analysis of effects from the use of peptides, Tag7 protein fragments, in a mouse model of DAD.

The results of measurements of cytokine levels in mouse plasma suggest that the investigated peptides of Tag7 may have a significant influence on the production of cytokines.

We started by investigating the expression of proinflammatory cytokines in the model with human PBMCs. Peptides of Tag7 influenced the initial stage of the immune response and changed the expression of mRNA of proinflammatory cytokines. We subsequently estimated the level of cytokines in plasma of ALI mice treated with studied peptides. The peptides had an impact on the secretion of cytokines, especially IFNγ. Alveolar macrophages are known to express TNFR1 and TREM-1 on their membranes and block signaling through TREM-1. Thus, the expression of proinflammatory cytokines in these cells is decreased [33]. It is suggested that the interaction of Tag7 peptides with the receptors plays a key role in reducing plasma IFNγ levels. In turn, a decrease in IFNγ correlates with lower levels of IL-4, which is also involved in immune regulation. Depending on cell type, IL-4 may have opposite effects, i.e., reduce or enhance IFNγ production [34].

The synergetic action of IFNγ and either IL-1α or TNFα resulted in strong induction of RANTES by human renal tubular epithelial cells [35]. The level of biologically active cell surface IL-15, which is involved in the regulation of host defense and inflammation, is also increased in response to IFNγ [36]. Finally, MIG [37] and IL-12p40 [38] are directly controlled by the IFNγ activity, and therefore all these signaling molecules may follow a systemic decrease of the IFNγ level. Another important physiological effect of Tag7 peptides is the decrease in the plasma level of LIF, which belongs to the IL-6 receptor family. It may be noted that the spectra of the activated cytokines differ between PBMCs and the mouse system. This could be due to a more complex system of the immune cell activation in the mouse and the involvement of different activating cells, as we have noted above in the manuscript.

Histological studies confirmed that the investigated peptides had an anti-inflammatory effect in the ALI model. As demonstrated, Tag7 N-terminal peptides effectively prevented the progression of the inflammatory process at the early stages of induced ALI. At later stages of ALI development, a more pronounced therapeutic effect was achieved with Tag7 protein C-terminal peptides, which protect the cells against TNFα-induced cell death.

The findings suggest that short peptides 17.1 and N1, which are fragments of innate immunity protein Tag7, can be used in the future as efficacious agents for the treatment or prevention of acute lung injury, e.g., in patients with COVID-19.

## 4. Materials and Methods

### 4.1. Cell Culture and Sorting

K562 cells were cultured in RPMI-1640 with 2 mM L-glutamine and 10% FCS (Invitrogen, Carlsbad, CA, USA). Human peripheral blood mononuclear cells (PBMCs) were isolated from the total leukocyte pool of healthy donors as described in [39] and cultured at a density of 4 × 10^6^ cells/mL in the same medium with LPS from Salmonella enterica (Sigma-Aldrich, Burlington, CA, USA) (100 ng/mL), Tag7 (10^−9^ M) or Hsp70 (10^−9^ M) for 6 days. Separation of lymphocyte subpopulation from PBMCs was performed using monocyte negative isolation magnetic bead kits and sheep anti-rabbit Dynabeads (Dynal Biotech ASA, Oslo, Norway) according to the manufacturer’s protocol.

### 4.2. Proteins and Antibodies

Recombinant Tag7 and Hsp70 were prepared as described in [39]. The Pierce Chromogenic Endotoxin Quant Kit (Thermo Fisher Scientific, Waltham, MA, USA) detected no bacterial LPS in the recombinant protein preparation. sTREM-1 was obtained according to [40]. Peptides of Tag7, inhTLR4 (Santa Cruz Biotechnology, USA; 1 μM), abTNFR1 (Santa Cruz Biotechnology, Santa Cruz, CA, USA; 1:500) or LP17 (LQVTDSGLYRCVIYHPP, 10^−9^ M) were added 1 h before lymphocyte treatment with activating agents.

### 4.3. Peptides

Protein Tag7 was hydrolyzed at 37 °C for 5 h at a 1:10 trypsin/protein ratio (*w/w*) in 50 mM (NH_3_)HCO_3_ (pH 8.0). The hydrolysate was then separated on a Superdex Peptide column. Aliquots of each fraction (1 mL) were applied to the PBMCs before treatment with Tag7. Peptides were synthesized as described in [16].

### 4.4. Affinity Chromatography, Immunoadsorption and Immunoblotting

Column with CNBr-activated Sepharose 4B (GE Healthcare, Chicago, IL, USA) conjugated with sTREM-1 was prepared following the manufacturer’s protocol. N1 peptide was biotinylated according to the protocol from [41]. Biotinylated N1 (biotin-N1) peptide was loaded onto the sTREM-1–Sepharose column. Excess peptide was washed from column with PBS containing 0.5 M NaCl and PBS alone. Peptide elution was conducted with 0.25 M triethylamine (TEA), pH 12. The eluted material was resolved by tricine–SDS-PAGE and blotted onto a nitrocellulose membrane [42]. HRP-conjugated streptavidin (GE Healthcare, Chicago, IL, USA; 1:15,000; 1 h) was used for detection. The results were visualized using ECL Plus Kit (GE Healthcare, Chicago, IL, USA) according to the manufacturer’s protocol. Chemiluminescence was detected using iBright (Thermo Fisher Scientific, Waltham, MA, USA). Monocytes (10^6^ cells) were incubated with biotin-N1 peptide (10^−8^ M) in the presence of BS^3^ (Thermo Fisher Scientific, Waltham, MA, USA), lysed in RIPA buffer (Sigma-Aldrich, Burlington, CA, USA) and purified using Dynabeads (M-280 Sheep Anti-Rabbit IgG; Dynal Biotech ASA, Oslo, Norway) conjugated with anti-TREM-1 antibodies (Abcam, Cambridge, UK) according to manufacturer’s protocol. This material was resolved by 10% PAGE followed by Western blotting.

### 4.5. Cytotoxicity Assays

For cytotoxic tests, target cells were cultured in 96-well plates (6 × 10^4^ cells per well) and mixed with lymphocytes at a 1:20 ratio. Cytotoxicity was measured after 24 h incubation. The inhibition test was conducted with peptides LP17, N1, 17.1 and 17.1a at a concentration of 10^−9^ M. Cytotoxic activity of lymphocytes was detected with a Cytotox 96 assay kit (Promega, Madison, WI, USA) according to the manufacturer’s protocol.

### 4.6. RT-PCR

RNA was isolated from the PBMCs or the fraction of monocytes (purified by magnetic cell separation) after they were treated with LPS (100 ng/mL) for 12 h. The inhibition test was conducted with peptides LP17, N1, 17.1 and 17.1a at a concentration of 10^−6^ M; TLR4 inhibitor (1 µM); and abTNFR1 (1:500). Monocytes or PBMCs (3 × 10^6^ cells per sample) were lysed in 500 µL of TRI Reagent (Sigma-Aldrich, Burlington, CA, USA) according to the manufacturer’s protocol. RNA measurement was conducted with NanoDrop (Thermo Scientific, Waltham, MA, USA), and equal amounts of RNA were used (1.1 µg). To estimate RNA degradation, electrophoresis was used. The synthesis of cDNA was performed with oligo (dT) primers (Eurogen, Moscow, Russia). The products were used for qPCR with primers for genes encoding RPLP0, GADPH, TNFα, IL-1β, IL6 and IFNγ. The levels of RPLP0 and GADPH mRNA were taken as a reference. The primers were as follows: RPLP0, forward: 5′-ACTGGAGACAAAGTGGGAGCC, reverse: 5′-CAGACACTGGCAACATTGCG; GAPDH, forward: 5′-CAACAGCGACACCCACTCCT, reverse: 5′-CACCCTGTTGCTGTAGCCAAA; IFNγ, forward: 5′-GGGTTCTCTTGGCTGTTACTG, reverse: 5′-TTCTGTCACTCTCCTCTTTCCA; TNFα, forward: 5′-CTTCTCCTTCCTGATCGTGC-3′, reverse: 5′-GCTGGTTATCTCTCAGCTCCA; IL-6, forward: 5′-GGAACAAGCCAGAGCTGTGC, reverse: 5′-TGCCGAAGAGCCCTCAGG; IL-1β, forward: 5′-GTACGATCACTGAACTGCACGC, reverse: 5′-CACGCAGGACAGGTACAGATTC.

Measurements at each point were made in at least three replications, and the mean value was calculated. Expression levels were quantified using the 2^ΔΔCt^ method.

### 4.7. The Design of Animal Experiment

ICR male mice with a mean weight of 40.2 ± 2.45 were used in the experiment. The animals were housed under the standard conditions of the Animal Breeding Facility, BIBCh, RAS (the Unique Research Unit Bio-Model of the BIBCh, RAS; the Bioresource Collection—Collection of SPF-Laboratory Rodents for Fundamental, Biomedical and Pharmacological Studies was supported by the Ministry of Science and Higher Education of the Russian Federation, contract No. 075-15-2021-1067), accredited at the international level by AAALAC.

All experiments and manipulations were approved by the Institutional Animal Care and Use Committee (No. 740/20, 17/02/20). The animals were randomly divided into seven groups (six animals in each group, *n* = 6): Group 1, intact animals without any treatment; Group 2, induction of DAD amid administration of 100 µL of normal saline; Group 3, induction of DAD amid administration of 120 µg of peptide 17.1; Group 4, induction of DAD amid administration of 120 µg of peptide 17a; Group 5, induction of DAD amid administration of 120 µg of peptide N1; Group 6, induction of DAD amid administration of 120 µg of peptide N2. The observation of animals was performed on days 7, 14 and 30.

In the model of induced lung damage, to induce an acute lung injury, a single instillation in the left lung was made with the compound containing 100 µL (1 mg/mL) of the LPS from Salmonella enterica and 100 µL (50 µg/mL) of α-galactosylceramide. Intubation of the left main bronchus was performed with an IV catheter 20G. Propofol, an ultra-short-acting sedative–hypnotic, was used for premedication of tracheal intubation. It was injected intravenously as a bolus in the lateral vein of the tail in a dose of 20 mg/kg.

### 4.8. Multiplex Assay

The level of plasma cytokines was measured using a MILLIPLEX MAP Mouse Cytokine/Chemokine Magnetic Bead Panel (Merck-Millipore, Burlington, MA, USA) according to the manufacturer’s instructions in mice with DAD 3, 10 and 24 h after syndrome induction.

### 4.9. Histological Studies

Pharmacological effects of the peptides and saline solution on DAD were assessed on days 7, 14 and 30.

After the animals were euthanized, the lungs were extracted and filled with 10% neutral formalin solution. The tissue specimens were rinsed in running water, dehydrated in an ascending alcohol series and embedded in paraffin. Then, 4–5 µm paraffin sections were stained with hematoxylin and eosin and examined by ordinary light microscopy using an AxioScope A1 (Carl Zeiss, Oberkochen, Germany). Microphotographs of the histological sections were made with Axiocam 305 color high-speed camera (Carl Zeiss, Oberkochen, Germany) and the software ZEN 2.6 lite (Carl Zeiss, Oberkochen, Germany). Histological examination included the assessment of the following morphological characteristics: peribronchial and perivascular infiltration with the mononuclear cells, infiltration of alveolar walls and ducts with the mononuclear cells, sites of pulmonary collapse and the presence/absence of necrotic foci.

The extent of different inflammatory manifestations in the lungs was evaluated using a semiquantitative scoring scale: 0—none (within the normal range); 1—minimal; 2—mild; 3—moderate; 4—severe, tissue alterations are noticeable, but there is a potential for increase in severity; 5—very severe, the maximal extent of alterations, characterizing the total lobe injury.

### 4.10. Statistical Analysis

Data are presented as mean ± standard deviation. All experiments were repeated at least three times. Differences between treatment and control were tested for significance with SigmaPlot software (Systat Software Inc, Berkshire, UK), using Student’s *t*-test for experiments on cell treatment with a single agent and one-way ANOVA for experiments on cell treatment with two or more agents (see individual figure legends).

## Figures and Tables

**Table 1:** Scoring scale table of peribronchial and perivascular infiltration with the mononuclear cells, infiltration of alveolar walls and ducts with the mononuclear cells and sites of pulmonary collapse of male ICR mice on the 7th, 14th and 30th days after induction of DAD in presence of peptides 17.1, N1, N2 and 17.1A or without treatment (*n* = 6 for each group).

**Days after Induction of DAD**	**Experimental Groups**	**Peribronchial Infiltration with the Mononuclear Cells**	**Perivascular Infiltration with the Mononuclear Cells**	**Infiltration of Alveolar Walls with the Mononuclear Cells**	**Infiltration of Alveolar Ducts with the Mononuclear Cells**	**Sites of Pulmonary Collapse**
		**Scores**				
7	Intact (no DAD)	0	0	0	0	0
	DAD (no treatment)	4	4	4	4	5
	DAD + Peptide 17.1	4	4	4	4	5
	DAD + Peptide N1	3	3	4	4	3
	DAD + Peptide N2	3	3	4	4	4
	DAD + Peptide 17.1A	4	4	4	4	5
14	Intact (no DAD)	0	0	0	0	0
	DAD (no treatment)	3	5	4	4	3,5
	DAD + Peptide 17.1	3	4	4	4	3,5
	DAD + Peptide N1	3,5	4	4	4	4
	DAD + Peptide N2	4	4	4	4	3
	DAD + Peptide 17.1A	3	4	4	4	4
30	Intact (no DAD)	0	0	0	0	0
	DAD (no treatment)	4	5	4	4	4
	DAD + Peptide 17.1	3	4	3	3	2
	DAD + Peptide N1	4	5	4	4	3
	DAD + Peptide N2	4	4	4	4	4
	DAD + Peptide 17.1A	4	4	4	4	4

## Supplementary Materials

The following are available online at www.mdpi.com/1422-0067/222/1/1213/s1.
