# Supplementary material for: Protein PGLYRP1/Tag7 Peptides Decrease the Proinflammatory Response in Human Blood Cells and Mouse Model of Diffuse Alveolar Damage of Lung through Blockage of the TREM-1 and TNFR1 Receptors
Source: Int J Mol Sci. 2021 Oct 18;22(20):11213. doi: 10.3390/ijms222011213 (PMC8538247; doi:10.3390/ijms222011213)
Supplement: Supplementary file 1 [file ijms-22-11213-s001.zip › ijms-1397616-supplementary.pdf]

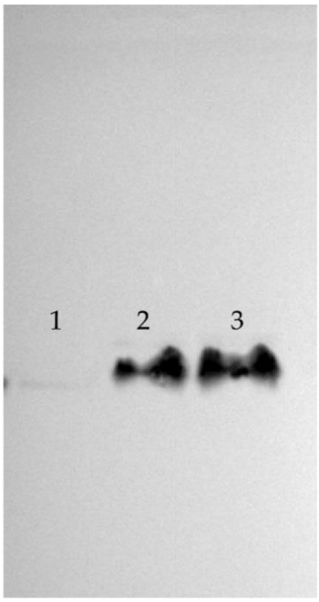

Supplemental Figure S1. Binding biotin-N1 with sTREM-1, immobilized on CNBr-Sepharose; 1-washing before elution, 2-elution of N1 peptide,3 – control N1 peptide. The resulting material was analyzed with Tricine-SDS-PAGE followed by WB.

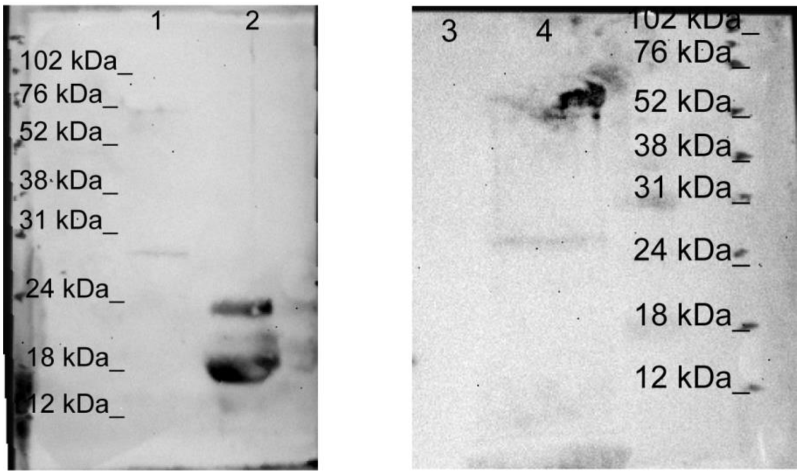

Supplemental Figure S2. Binding biotin-N1 with sTREM-1 on monocytes. Monocytes incubated with biotin-N1 and treated with crosslinker BS_3. Cells lysed and purified with Dynabeads, conjugated with anti-TREM-1 antibodies (1) and (4); biotin sTREM-1 purified with Dynabeads, conjugated with anti-TREM-1 antibodies (2); untreated monocytes lysed and purified with Dynabeads, conjugated with anti-TREM-1 antibodies (3). The resulting material was analyzed by SDS electrophoresis followed by Streptavidin-HRP Western blotting.

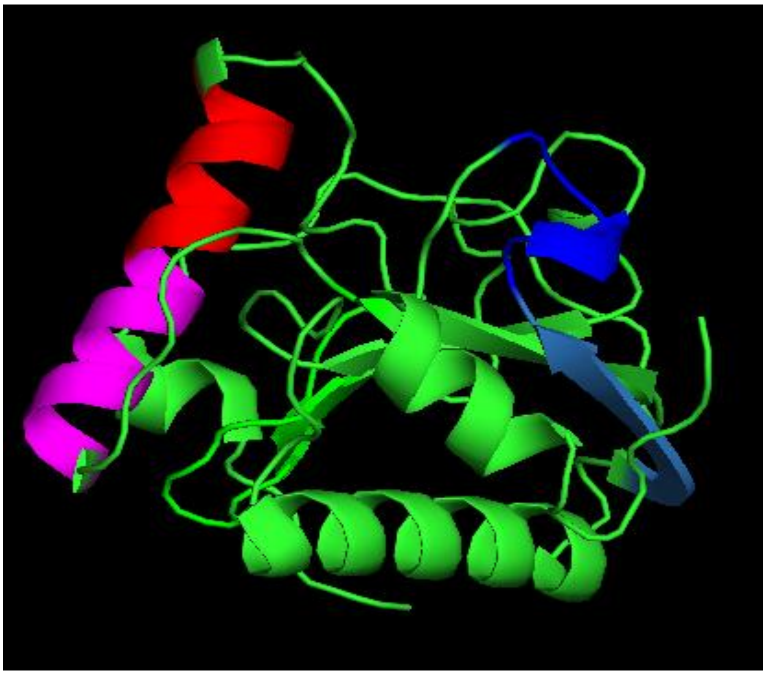

Supplemental Figure S3. A 3D structure of Tag7 (PDB-1YCK). N1 – red, N2 – pink, 17.1A – light blue is part of 17.1 – all blue.
